# Enhanced Spinal Therapy: Extracorporeal Shock Wave Therapy for the Spine

**DOI:** 10.7759/cureus.11200

**Published:** 2020-10-27

**Authors:** Brian Fiani, Cyrus Davati, Daniel W Griepp, Jason Lee, Elisabeth Pennington, Christina M Moawad

**Affiliations:** 1 Neurosurgery, Desert Regional Medical Center, Palm Springs, USA; 2 Medicine, New York Institute of Technology, Old Westbury, USA; 3 Medicine, Chapman University, Orange, USA; 4 Medicine, University of Illinois at Urbana-Champaign, Champaign, USA

**Keywords:** extracorporeal shock wave therapy, eswt, shockwave, spinal cord injury, disc regeneration, scoliosis, osteoporosis, cervical spondylosis, coccydynia, non-invasive

## Abstract

Extracorporeal shock wave therapy (ESWT) is a non-invasive therapeutic method used for pain management and muscle strength improvement through the use of shock waves. In vitro studies have demonstrated that shockwave therapy induces fluctuation in redox reaction regulation and increases in Mitogen-Activated Protein Kinase (MAPK) signal transduction pathways, stimulating increased gene expression in the nucleus. ESWT has also been shown to upregulate angiogenesis and growth factors through activation of endothelial nitric oxide synthase (eNOS) and vascular endothelial growth factor (VEGF). The use of ESWT in the treatment of various musculoskeletal disorders was widely adopted throughout Europe, South America, and Asia before being introduced in the United States in 2000. Within the past 20 years, the clinical application of ESWT in the treatment of musculoskeletal and bone disorders has grown. This paper provides a comprehensive narrative review of applications and outcomes of ESWT in clinical spinal pathology and assesses reported efficacy as it relates to the pathology. A review of the literature yielded studies describing the use of ESWT in degenerative osteoporotic neuro-spinal pathology, heterotopic ossification due to spinal cord injury, cervical spondylosis, scoliosis, sacroiliitis, and coccydynia. The efficacy of ESWT as an adjunct treatment in patients with spinal cord pathologies varied with the specific pathology, however, all pathologies discussed in this review provided evidence of potential benefits with minimal adverse effects. While the use of ESWT for pain management has widely been established, further literature should aim to identify the long-term benefits of ESWT.

## Introduction and background

Extracorporeal shock wave therapy (ESWT) is a non-invasive therapeutic method used for pain management and muscle strength improvement through the use of shock waves to promote revascularization [1]. The concept of ESWT first emerged from observations of osteoblastic response patterns during animal studies in the mid-1980s [2]. The use of ESWT in the treatment of various musculoskeletal disorders was widely adopted throughout Europe, South America, and Asia before being introduced in the United States in 2000 [2]. Initially, the FDA (Food and Drug Administration) approved the use of specific shockwave devices for the treatment of plantar fasciitis in 2000, followed by approval for the treatment of lateral epicondylitis of the elbow in 2003 [2]. Within the past 20 years, clinical applications of ESWT in the treatment of musculoskeletal and bone disorders have grown to include, but are not limited to, use in lateral epicondylitis of the elbow, patellar tendinopathy (Jumper’s knee), Achilles tendinopathy, calcifying tendinitis of the shoulder, non-union and delayed union of long bone fracture, and avascular necrosis of the femoral head [2]. More recently, the exploration of clinical applications beyond non-musculoskeletal diseases has included the use of ESWT in the treatment and management of pain for chronic diabetic foot ulcers, ischemic heart disease, complex regional pain syndrome, osteoarthritis of the knee, malignant cells, gene therapy, and spinal fusions [2].

While the potential of ESWT in the treatment of a wide variety of conditions has been acknowledged, current FDA approved indications of use remain limited, suggesting the need for further research discussing the efficacy of ESWT in alternative indications. The objective of this paper is to provide a comprehensive review of potential indications for ESWT in spinal cord pathology and assess efficacy as it relates to the individual pathology. Efficacy will be determined through the analysis of outcomes from currently published studies on ESWT as a treatment protocol in degenerative osteoporotic neuro-spinal pathology, heterotopic ossification due to spinal cord injury, cervical spondylosis, scoliosis, sacroiliitis, and coccydynia.

## Review

Mechanism of ESWT therapy

Extracorporeal shockwaves are rapid oscillations of pressure waves that can travel through various mediums. A shockwave is a non-linear pressure with a duration of 10 microseconds. There is a positive phase, characterized by high-pressure waves hitting a tissue interface that allow penetration or reflection. During the negative phase, the accelerated pressure causes the tissue layers to cavitate, inducing an air bubble formation and produces a second shock wave [3]. The energy that is produced fluctuates in its size and its volume depending on the structural composition of the target tissue, allowing proper pressure application in damaged tissue locations. Wave energy transfer involves the absorption and refraction of induced waveforms allowing for transmission to tissue. The high energy induced by shockwave therapy causes an alteration in cell structure, while lower energy dissipation induces changes in the cell membrane and cytoplasmic organelles, thus stimulating the nucleus [4]. In vitro studies demonstrate that shockwave therapy induces fluctuation in redox reaction regulation and increases in extracellular signal-related kinases (ERK) signal transduction pathways, stimulating increased gene expression in the nucleus. Through repeated experimentation is was shown that application of ESWT upregulates angiogenesis and growth factors through activation of endothelial nitric oxide synthase (eNOS) and vascular endothelial growth factor (VEGF) [3]. The increase in nitric oxide formation from eNOS activation promotes differentiation of human osteoblasts. Upregulation of VEGF stimulates wound healing through collagen deposition and epithelization. Increase in neovascular formation is seen within one week of ESWT application and plateau around four weeks [3]. ESWT has also shown to induce molecular expression of proliferating cell nuclear antigen (PCNA) and bone morphogenetic protein-2 (BMP-2), both essential for cell replication along with von Willebrand factor​​​​​​​ (vWF) for increasing clotting demands [3]. Through triggering osteogenic transcription factors, post-operative fracture healing is accelerated.

Extracorporeal shockwave therapy is divided into focused shockwave therapy (FSWT) and radial shockwave therapy (RSWT). In focused shockwave therapy, a pressure field converges into a selected focus frame at specific depths within body tissue. There are three different shockwaves used, including electrohydraulic, electromagnetic, and piezoelectric which are all waves generated in water [5]. Water acoustic wave transduction is very similar to biologic tissue, allowing for comparable signal transduction. Through this engineering method, waves are transferred much easier throughout the body. In radial shockwave therapy, a pressure wave is generated through accelerating a projectile through compressed air until it reaches an applicator, causing it to generate waves throughout the body [5]. In radial therapy, the shockwave dispersion is more superficial while focused therapy penetrates deeper into body tissue. Both modalities of treatment generate different therapeutic effects through pain relief and tissue regeneration.

Applications

Osteoporosis

Osteoporosis commonly affects the elderly and post-menopausal women and has multiple clinical presentations in the spine, most commonly compression fractures of the vertebral body and resultant radiculopathies [6]. While the application of ESWT for treatment of osteoporosis has been studied extensively, there are few studies that describe its use for osteoporosis in the spine [5]. Several studies were identified as describing the use of ESWT for lower back pain, however, results and reported utility of treatment centered around reducing myofascial pain in the lumbar region and the trapezius in the neck region [7]. Review of the literature revealed only one study that described the use of ESWT that measured outcomes in the osteoporotic lumbar spine. In this study, bone mineral density (BMD) of the lumbar spine was assessed through dual-energy X-ray absorptiometry (DEXA) scan [8].

Shi et al. designed a randomized control trial (RTC) of 64 postmenopausal patients with a known history of osteoporosis with two treatment groups (n=20,21) and one control (n=23). Treatment groups were low energy flux density ESWT (0.15 mJ/mm2, 3 Hz, total 4000 impulses) and high energy flux density ESWT (0.28 mJ/mm2, 4 Hz, total 4000 impulses). These were applied by a Dornier Compact Delta II Emitter (Dornier MedTech GmbH, Weßling, Germany) with penetration depth and focus diameter of 15 and 4 mm, respectively (Table 1). Bone mineral density was measured at the lumbar spine, femoral neck, tibial tuberosity, and left hip pre-treatment and three, six, and 12 months post-treatment initiation. Interestingly, the ESWT therapy was applied directly to only the hip joint for the duration of treatment. At 12 months post-intervention, a significant increase in BMD (p <0.01) was observed at all four locations, including the lumbar spine. At the lumbar spine specifically, low energy flux density ESWT significantly increased BMD over the high energy flux density group, while both were also showed significantly improved BMD over control (Table 2). Increase in BMD in the spine is significant because it directly counteracts the degenerative changes occurring and can potentially prolong the asymptomatic period of vertebral body compressive changes and radicular symptoms. Some of the notable limitations in this study include a small sample size of treatment groups and lack of variation in energy flux density (low vs. high). Despite limitations, this study demonstrates the osteogenic effects of ESWT that should be considered in degenerative osteoporotic neuro-spinal pathology.

**Table 1:** Summary of ESWT treatment characteristics. ESWT: extracorporeal shock wave therapy

Study	Emitter Used to Apply ESWT	Energy Flux Density (mJ/mm^2^)	Frequency (Hz)	Total Impulse	Site of ESWT Application
Shi, 2017 [8]	Dornier Compact Delta II	0.15 - 0.28	3 - 4	4000	Hip region
Jeon, 2019 [9]	Dornier Aries AR2	0.056 - 0.068	3	4000	Hip joint
Li, 2020 [10]	n/a	0.06	8	4000	Hip joint
Lin, 2015 [11]	F10G4 Richard Wolf GmbH	0.27	1 - 8	2000	Cervical spine
Weiss, 2013 [12]	Storz Medical Masterpuls MP 100	n/a	n/a	2000	Thoracic spine
Weiss, 2017 [13]	Storz Medical Masterpuls MP 100	n/a	n/a	2000	Thoracic spine
Moon, 2017 [14]	Dornier Medtech Aries	0.09-0.25	3	2000	Sacroiliac joint
Marwan, 2014 [15]	n/a	0.2	n/a	3000	Coccyx
Lin, 2015 [16]	BTL-5000 radial	0.12 – 0.18	5	2000	Coccyx
Haghighghat, 2016 [17]	n/a	0.09	21	3000	Coccyx
Marwan, 2017 [18]	n/a	0.2	n/a	3000	Coccyx
Aydin, 2020 [19]	Physiomed Elektromedizin AG	0.2	n/a	3000	Coccyx

**Table 2:** Summary of patient treatment applications and outcomes reported in this review. NPS – numerical pain scale; VAS – visual analog pain scale; ODI – Oswestry disability index; NDI – neck disability index; ONL CSA – ossification of nuchal ligament cross-sectional area; NHO CSA – neurogenic heterotopic ossification cross-sectional area; FFD – finger to floor distance; ATR - angle of trunk rotation; LD – lateral deviation; SR – surface rotation; DEXA: dual energy X-ray absorptiometry; SF-36: 36-Item Short Form Health Survey; ESWT: extracorporeal shock wave therapy

Study	Pathology	# of Patients Treated in Study		Outcomes of Interest	Treatment Regimen	Duration of ESWT Effect or Follow-up
Shi, 2017 [8]	Osteoporosis		41	BMD (DEXA scan)	1 session total	1 year
Jeon, 2019 [9]	Heterotopic ossification due to spinal cord injury		1	NHO CSA, VAS	7x weekly 7 weeks	6 months
Li, 2020 [10]	Heterotopic ossification due to spinal cord injury		1	NHO CSA, VAS, ROM	3x weekly ~ 50 weeks	6.5 months
Lin, 2015 [11]	Cervical spondylosis, nuchal ligament calcification		40	ONL CSA, NDI, VAS, ROM	3x weekly 6 weeks	3 months
Weiss, 2013 [12]	Scoliosis		15	LD, SR, kyphosis, lordosis	1 session total	3-5 days
Weiss, 2017 [13]	Scoliosis		1	FFD, ATR	5x weekly 5 weeks	3-5 days
Moon, 2017 [14]	Sacroiliitis		15	ODI, NPS	1 session total	4 weeks
Marwan, 2014 [15]	Coccydynia		2	NPS, VAS	1x weekly 3 weeks	1 year
Lin, 2015 [16]	Coccydynia		21	ODI, VAS	3x weekly 4 weeks	2 months
Haghighghat, 2016 [17]	Coccydynia		10	VAS	4 sessions total	2 months
Marwan, 2017 [18]	Coccydynia		17	ODI, NPS, VAS	3 sessions total	6 months
Aydin, 2020 [19]	Coccydynia		34	VAS, SF-36	1x weekly until improvement	6 months

Spinal Cord Injury

A recent finding by Yahata et al. suggests that the use of low-energy ESWT following spinal cord injury stimulated VEGF and angiogenesis in neural cells and enhances angiogenesis in damaged neural tissue [20]. Furthermore, ESWT was found to have the neuroprotective effect of suppressing cell death and axonal damage, improving sensory and locomotor functions. While results in animal studies are promising, the use of ESWT in spinal cord injury is limited. There are currently no clinical studies or reported cases that have demonstrated the use of ESWT applied directly to the site of spinal cord injury. Instead, ESWT in spinal cord injury (SCI) thus far has been targeted towards treating pain due to neurogenic heterotopic ossification (NHO), which is pathologic ectopic bone formation in soft tissues that sometimes follows central nervous system damage [21, 22]. This often forms around the hip joint muscles, as in the two reported cases treated with ESWT, causing significant pain and hindrance of range of motion and mobility [23, 24]. Consequently, the physical ESWT treatment is not directed towards the spine but rather the site of ectopic ossification, often until the reduction of patient pain or return of joint mobility. Although Boxberg et al. [25] first described the use of ESWT for use in soft tissue ossification in 1996 and previous studies have reported use in traumatic brain injury NHO, uses of ESWT in spinal cord injury specifically are a new addition to the medical literature, as the first two reported cases of treatment were in 2019 and 2020 [9, 10, 25, 26].

Jeon et al. reported application of ESWT in a wheelchair-bound patient with complete C4 SCI according to American Spinal Injury Association (ASIA) Impairment Scale A with reported pain Visual Analog Scale (VAS, 0-10) of 8 in the right hip [9]. Following failed conservative treatment (nonsteroidal anti-inflammatory drugs (NSAIDs) + bisphosphonate), ESWT was applied to the hip region using Dornier Aries AR2** **(Dornier MedTech GmbH, Weßling, Germany) under ultrasound guidance. Application of mild energy flux density ESWT (0.056 -0.068 mJ/mm2. 3 Hz, total 4000 impulses) was administered seven times weekly for seven weeks. Although the size of NHO measured with radiograph was not reduced significantly, the patient’s VAS score was reduced to a 3, with the therapeutic effects lasting for six months. Li et al. described ESWT in a patient with a history significant for incomplete C8 SCIaccording to American Spinal Injury Association (ASIA) Impairment Scale D with reported pain VAS of 8 in the left hip [10]. Similarly, ESWT was applied (unspecified probe) with similar energy flux density ESWT (0.06 mJ/mm2. 8 Hz, total 4000 impulses), five times weekly for one year. The NHO reduced from 45x25mm to 18x16mm and VAS was reduced to 1 within 1.5 months of treatment initiation. In both cases, despite the varying degree of spinal cord injury, the outcome of interest (pain reduction due to NHO) was achieved through continued ESWT therapy.

Cervical Spondylosis

The nuchal ligament is an important structure that maintains lordotic alignment and stabilizes the head and cervical spine during movement [27, 28]. Ossification (or calcification) of the nuchal ligament (ONL) can form around the spinal processes of the cervical spine, contributing to cervical pathology [29, 30]. When the nuchal ligament is stiffened, the mechanical compressive forces on the cervical spine are accentuated with head movement, particularly on the soft cervical discs and nerve roots, which have specific neurological sequelae when untreated, particularly at the lower cervical segments. A study of patients with ONL by Tsai et al. showed that larger ONL was significantly associated with more stiffness in neck flexion and extension, worsened changes in the cervical neural foramen, more complex cervical spondylosis, and more severe radiculopathy [31]. Review of the literature demonstrated one study by Lin et al. where ESWT was recruited in a randomized control trial to treat 60 patients with cervical spondylosis and known nuchal ligament calcification [11].

In this study, prior to treatment, cross-sectional area (CSA) of ONL was measured with x-ray radiograph, range of motion​​​​​​​(ROM) was measured with a goniometer, neck disability was measured with neck disability index (NDI, 0-50) and pain was measured with VAS (0-10). All three study groups received hot packs and traction force to the cervical spine, while the two treatment groups received additional x-ray guided ESWT or musculoskeletal sonography guided ESWT. In both treatment groups, ESWT was applied using an F10G4 shockwave generator (Richard Wolf GmbH, Knittlingen, Germany) (0.27 mJ/mm2. 1-8 Hz, total 2000 impulses) to the patient’s flexed neck in a seated position, once weekly for six weeks. At three months following complement of treatment, ESWT groups showed significant improvement of NDI, VAS, and ROM over both the initial assessment and the conservative treatment control group (hot packs/traction). Throughout each of these outcome measures, musculoskeletal sonography guided ESWT had more favorable results compared with X-ray guided, showing a significant improvement over x-ray guided ESWT specifically in neck bending ROM. CSA was the only outcome measure that did not significantly change in any of the groups throughout the course of treatment or at follow up. This study demonstrated ESWT as an effective adjuvant treatment to achieve pain reduction, improved ROM, and lower NDI scores in patients with cervical spondylosis and calcification of the nuchal ligament.

Scoliosis

The first use of ESWT for the treatment of scoliosis was described by Weiss et al. in 2013 through a pilot study of 15 adolescent patients [12]. The rationale of ESWT treatment in this study was directed towards improving impairment of forward flexion (IFF), which is believed to occur over time due to functional tethering of the spinal cord [32, 33]. Tomaschewski et al. previously suggested IFF to be a precursor to structural spinal deformity and subsequent development of severe adolescent idiopathic scoliosis (AIS) [13, 34]. Furthermore, structural and neurological considerations of spinal cord tethering were reported in an MRI study of 81 scoliotic patients by Deng et al., which described lower spinal cord/vertebral length ratios, spinal cord deformity at the apical level of scoliotic curve, and deviation of the spinal cord in the central canal with lateral cord space on the convex side of spinal curvature [13, 33].

Thus, in the ESWT pilot study for scoliosis, therapy was localized to the lower thoracic segments where the “flatback contracture” was present and theoretically, where ESWT treatment was most likely to correct IFF and improve overall 3D deformity of the spine caused by tethering. A one-time ESWT treatment was administered to 15 girls with scoliosis (age of 14±1.1, range 12-15; cobb angle of 35.1±9.6, range 20-50) with a Storz Medical Masterpuls MP 100 system (Storz Medical AG, Tägerwilen, Switzerland) with trigger point applicator (unspecified energy flux density, unspecified frequency, total 2000 impulses). Outcomes were measured five minutes post-treatment and demonstrated consistent reduction of lateral deviation and surface rotation (desirable) as well as increased kyphosis and lordosis angles (desirable) when compared to pre-treatment values. Although effects were no longer present within a few days, Weiss et al. concluded that vertebral improvements post-intervention fit into the concept of functional tethering. The idea being that ESWT temporally improved spinal restriction that had occurred chronically over time due to continuous functional tethering on the spine [12, 32]. In 2017, Weiss et al. reported similar application of ESWT to treat a single 13-year-old girl with known scoliosis and a cobb angle of 24 degrees, however, once weekly for five weeks. ESWT was applied (same applicator setup) directly to the thoracic vertebra with the patient in the diagnostic position described by Tomaschewski (closed knee-chest) [13, 34]. This time, finger to floor distance (FFD) and angle of trunk rotation (ATR) were measured immediately pre and post-intervention each week. Finger to floor distance was chosen as it is strongly correlated with the mobility of the spinal cord [35, 36]. On average, FFD improved from 22.6 to 15.6cm (p<0.01) and ATR improved from 13 degrees to 10 degrees (p <0.05) following ESWT treatment, however, measurements consistently returned to initial values 3-5 days post-intervention each time, indicating long term effects of ESWT were not observed in either study.

Sacroiliitis

The sacroiliac joint is a diarthrodial joint that connects the axial skeleton to the lower extremities [37]. Disruption of the natural architecture of this joint by the way of trauma, infection, or inflammatory disease can lead to sacroiliac joint (SIJ) pain [37]. This classically manifests as localized pain just distal and medial to the posterior superior iliac spine​​​​​​​ (PSIS) with referred pain to the buttock, lower lumbar spine, and/or groin [37, 38]. SIJ pain has been reported to also affect the lower extremities in approximately 50% of patients, with the posterior and lateral thigh being the most common areas experiencing pain [39, 40]. SIJ pain is traditionally managed in a stepwise manner that begins with pharmacological treatment and physical therapy (stretching, mobilization activities, joint manipulation). Patients that do not receive adequate pain relief via these conservative treatment options may be additionally administered intra-articular corticosteroid injections. Although therapeutic injections have been shown to provide pain reduction and functional improvement of SIJ pain, they also carry risks that include infection, hematoma formation, vascular infiltration and corticosteroid associated side-effects [14]. As such, alternative treatment is recommended for patients that require greater than three injections in a six-month period or four injections in a year [37].

Moon et al. conducted a randomized sham-controlled trial that consisted of 25 patients with SIJ pain referred to the rehabilitation clinic at a university hospital [14]. The participants were divided into a treatment group (n=14) and a control group (n=11). The treatment group received a single session of ESWT comprised of 2,000 shocks at 3 Hz delivered by a Dornier MedTech Aries (Dornier MedTech GmbH, Weßling, Germany) probe set to the maximum energy level tolerated by the patient (0.09-0.25 mJ/mm2). The probe was oriented perpendicular to the posterior SIJ line and moved up and down slowly using ultrasound gel as a coupling agent. The control group alternatively received a single session of sham ESWT comprised of minimal energy pulses (0.03 mJ/mm2) delivered by the same Dornier MedTech Aries probe, but with the probe oriented parallel to the posterior SIJ line. Additionally, the ESWT machine in the control group produced an audible noise in conjunction with every shockwave delivery to improve the sham design.

The intensity of pain and understanding of function was evaluated using the numeric rating scale (NRS) and Oswestry disability index (ODI), respectively, both prior to treatment and four weeks after. At four weeks post-treatment, NRS scores in the ESWT group showed significant improvement compared to baseline with no significant improvement observed in the control group. The ODI scores in contrast, did not show any significant post-treatment changes in both groups. However, the ODI score in the ESWT group did exhibit a notable trend towards improvement at both one and four weeks post-treatment as compared to baseline. Finally, there was a significant difference between the NRS scores in the ESWT and control group at four weeks post-treatment but no significant difference in ODI scores. It is hypothesized that ESWT improves pain by either damaging, and thus suppressing pain-conducting unmyelinated fibers, and/or by inducing neovascularization and tissue regeneration of the ligamentous structure that forms the posterior aspect of the SIJ [41-44]. Thus, applying ESWT to the posterior SIJ line (as performed in this study), can promote stability and limit motion that precipitates SIJ pain. Despite limited sample size and lack of long-term follow-up, the significant improvement in SIJ pain coupled with the absence of patient-reported side effects, such as skin redness, petechial bruising, or subcutaneous hematoma after ESWT, demonstrates that ESWT should be considered for the treatment of SIJ pain.

Coccydynia

Coccydynia occurs due to inflammation of the coccyx, or its joint with the sacral vertebra, causing pain most commonly experienced upon moving from a seated to standing position [19, 45]. The pathophysiology is most commonly attributed to coccygeal instability, subluxation, or trauma and it is five times more likely to occur in women [16, 17]. The coccyx is associated with the fifth sacral and coccygeal nerve roots and the terminal sympathetic plexus [19]. Additionally, the filum terminale, also called the coccygeal ligament, inserts onto the first coccygeal segment, which is of particular neuroanatomical significance in clinical pathologies involving the spinal cord [45-49]. Conservative management of coccydynia includes NSAIDs, ligament massage, manual manipulation for malalignment, steroid and anesthesia injections, or physical therapy with interferential current or shortwave diathermy [16, 20]. ESWT has recently emerged for the treatment of coccydynia and has been proposed as an effective treatment for relieving pain and improving quality of life with promising outcomes reported [15-18, 19].

Analysis of current published studies suggests that Marwan et al. was the first to describe the use of ESWT for coccydynia, reporting favorable results in treating two patients [15]. Over the past five years, four additional studies explored ESWT use with larger sample sizes (range of 10-34 patients). The ESWT treatment protocols in each study were similar as each study applied moderate energy flux density (ranging from 0.09 - 0.2 mJ/mm2, total 2000 - 3000 impulse) to the point of maximal coccyx tenderness, once weekly for three to four consecutive weeks. Marwan et al. and Aydin et al. both applied ESWT with patients in a lateral lying position with flexed knees, while Lin et al. applied therapy with patients prone and knees flexed [16, 18, 19]. Concerning the duration of effect of ESWT, Lin et al. and Haghighat et al. demonstrated that effects were observed for at least two months, while Marwan et al. and Aydin et al. found it to be as long as six months [16-19]. The first two cases reported by Marwan et al. in 2014 had the longest duration of follow up of one year, with favorable results [15]. Haghighat et al. was the only investigator to report findings that potentially suggest the effects of ESWT began to reverse. In this study, VAS pain scores were lowest at two months post-treatment initiation and had increased at seven months follow up, however, not to pre-treatment levels and were still significantly lower [17]. A notable limitation of these studies is the lack of a control group, with the exception of the randomized control trial by Lin et al. which compared ESWT to combined physical therapy with interferential current or shortwave diathermy treatment [16]. In this study, ESWT was shown to be superior to the physical therapy treatment group with regard to pain reduction, grade of improvement in disability, and subjective satisfaction after two months [16]. The authors of this review believe that these five studies on coccydynia show strong support for continued use of ESWT in coccydynia, however, continued research into the duration of ESWT effect should be pursued.

## Conclusions

The use of ESWT in patients with a known history of osteoporosis may be indicated, as suggested by the increased BMD levels and interpretation of decreased degenerative changes in the 12-month intervention following treatment. ESWT for the treatment of pain secondary to heterotopic ossification due to spinal cord injury was found to be effective through continued use of ESWT, as indicated by decreased VAS scores reported by patients and decreased NHO measurements. Similarly, the treatment of pain secondary to cervical spondylosis also suggested the effectiveness of ESWT, as indicated by decreased patient-reported pain levels. Furthermore, the use of ESWT as an adjunct treatment in patients with cervical spondylosis and calcification of the nuchal ligament was associated with improved ROM and lower NDI scores, suggesting the benefits of ESWT are not limited to pain reduction. Despite initial improvements as indicated by improved finger to floor distance (FFD) 22.6cm to 15.6cm (p <0.01) and angle of trunk rotation (ATR) 3 degrees to 10 degrees (p <0.05) following ESWT in patients with scoliosis, the long-term benefits of ESWT for patients with scoliosis was not established due to consistent benefit reversal three to five days post-intervention. In patients with sacroiliitis, significant improvements in SIJ pain and the absence of patient reported side effects following ESWT intervention indicate ESWT may be considered for the treatment of SIJ pain, however further research is warranted due to limited sample size and lack of long-term follow up. Despite decreased VAS pain scores and subjective satisfaction in patients with coccydynia following ESWT treatment, further research is warranted to establish the long term benefits of use due to findings suggesting the reversal of the effects of ESWT and limitations of the studies, including a lack of a control group.

The efficacy of ESWT as an adjunct treatment in patients with spinal cord pathologies varied with the specific pathology, however, all pathologies discussed in this review provided evidence of potential benefits with minimal adverse effects. While the use of ESWT for pain management has widely been established, further literature should aim to identify alternative benefits, including the role of ESWT in bone healing, and to establish the long-term benefits of ESWT.
